# Coverage of policies to improve antimicrobial stewardship in human medicine in low and middle income countries: results from the Global Survey of Experts on Antimicrobial Resistance

**DOI:** 10.1186/s12889-024-19542-2

**Published:** 2024-08-23

**Authors:** Kyaw Zay Ya, Mark J. Lambiris, Gillian A. Levine, Fabrizio Tediosi, Günther Fink

**Affiliations:** 1 Department of Epidemiology and Public Health, Swiss Tropical and Public Health Institute, Kreuzstrasse 2, Allschwil, 4123 Switzerland; 2 University of Basel, Basel, Switzerland; 3 Health Economics Facility, Department of Public Health, University of Basel, Basel, Switzerland; 4 Institute of Pharmaceutical Medicine (ECPM), University of Basel, Basel, Switzerland

**Keywords:** Antimicrobial resistance, Antibiotics, Policies, Legislation, Low and middle income countries

## Abstract

**Background:**

Antimicrobial resistance (AMR) constitutes a major threat to global health. While antimicrobial misuse or overuse is one of the main drivers for AMR, little is known about the extent to which antibiotic misuse is due to a lack of national government-led efforts to enforce rational use in low and middle-income countries (LMICs).

**Methods:**

To assess antimicrobial stewardship and national implementation measures currently in place for optimizing antimicrobial use and for slowing the spread of AMR, we invited public health experts from 138 LMICs to participate in a Global Survey of Experts on Antimicrobial Resistance (GSEAR). Key coverage measures, as reported by experts, were compared across countries and also juxtaposed with estimates collected in the 2020-21 World Health Organization-organized Tripartite AMR Country Self-Assessment Survey (TrACSS).

**Results:**

A total of 352 completed surveys from 118 LMICs were analysed. Experts in 67% of the surveyed countries reported a national action plan (NAP) on AMR, 64% reported legislative policies on antimicrobial use, 58% reported national training programs for health professionals, and 10% reported national monitoring systems for antimicrobials. 51% of LMICs had specific targeted policies to limit the sale and use of protected or reserve antibiotics. While 72% of LMICs had prescription requirements for accessing antibiotics, getting antibiotics without a prescription was reported to be possible in practice in 74% of LMICs. On average, country efforts reported in TrACSS were substantially higher than those seen in GSEAR.

**Conclusions:**

In many LMICs, despite the existence of policies aimed at slowing down the spread of AMR, there are still significant gaps in their implementation and enforcement. Increased national efforts in the areas of enforcement and monitoring of antibiotic use as well as regular monitoring of national efforts are urgently needed to reduce inappropriate antibiotic use in LMICs and to slow the spread of AMR globally.

**Supplementary Information:**

The online version contains supplementary material available at 10.1186/s12889-024-19542-2.

## Introduction

Antimicrobial resistance (AMR) poses an urgent threat to global health [1–3]. According to the most recent estimates, bacterial AMR was associated with 4.95 million deaths in 2019, with a particular high burden in Sub-Saharan Africa and South Asia [4]. In 2019, methicillin-resistant *Staphylococcus aureus* alone caused 100,000 deaths and a loss of 3.5 million disability-adjusted life years (DALYs) globally [4]. Many deadly pathogens have developed resistance to life-saving medicines, including multi-drug-resistant (MDR) tuberculosis, third-generation cephalosporin-resistant *Escherichia coli (E coli*), carbapenem-resistant *Acinetobacter baumannii*, fluoroquinolone-resistant *E coli*, carbapenem-resistant *Klebsiella pneumoniae*, and third-generation cephalosporin-resistant *Klebsiella pneumoniae*, all of which pose major threats to human health today [4].

One of the main drivers of increased AMR is the use, misuse or overuse of antimicrobials globally [4–6]. This is particularly true for Low and Middle Income Countries (LMICs), where overuse seems particularly common [7–10]. Over the past 20 years, estimated global antibiotic consumption rates have increased from 9.8 defined daily doses (DDD) per 1000 population per day in 2000 to 14.3 DDD per 1000 per day in 2018 globally [11]. Even though the development of AMR is to some extent inevitable, the emergence of antibiotic-resistant microbes can be slowed by reducing the overuse and misuse of antimicrobials as well as the resulting selection pressure on resistant bacteria [12].

The urgent need for global and concerted action to address AMR was recognized during the World Health Assembly 2015, where all countries agreed to develop and implement National Action Plans (NAPs) to address rising AMR [13, 14]. Relatively little data is currently available on the extent to which governments have actually implemented stewardship programmes and enforced policies to reduce antibiotic overuse and misuse, thereby potentially slowing AMR. [12, 15–17]. Recently, a systematic analysis of NAPs on AMR in 114 countries revealed considerable variations in national efforts to control AMR, which may not be proportionate to the scale and severity of the problem [18, 19].

To the best of our knowledge, the largest currently available database on specific national policies to address AMR globally is the Tripartite AMR Country Self-Assessment Survey (TrACSS) and published NAPs [14, 20]. Relatively little is known about the existence of legislative policies, policy enforcement or programs to optimize antimicrobial use and address AMR globally [15].

The four organizations promoting TrACSS include the United Nations Environment Programme (UNEP), the Food and Agriculture Organization (FAO), World Health Organization (WHO), and the World Organization for Animal Health (WOAH, formerly OIE). These four organizations collectively form the Quadripartite Technical Group on Integrated Surveillance of antimicrobial use and resistance, [14, 20, 21] and replace the previous Tripartite Collaboration for Antimicrobial Resistance Surveillance supported by the WHO, FAO and WOAH [14, 20]. TrACSS have been conducted since 2016 and collect data on a range of antimicrobial stewardship efforts, including implementation of NAPs, legislation on antimicrobial access, efforts to raise awareness of AMR, and training efforts to improve appropriate antibiotic use; as well as national antimicrobial use, monitoring, and surveillance systems [14]. Quality assurance of antimicrobials [22], the prevalence of counterfeit drugs in the market [23], and sufficient political interest and commitment are key areas of concern in LMICs [5, 17, 24]. However, global data on these topics are limited.

Existing evidence suggests that the implementation of certain essential medicines policies has the potential to effectively reduce the use of antimicrobials and thereby combat AMR, particularly in South East Asia [25–28]. In the current landscape of available policy analyses, reviews and surveys, there is evidence on addressing antimicrobial use from a social science, policy analyses to reduce antimicrobial overuse, and reviews to monitor global commitments [15, 29]. However, there is a notable gap in the availability of comprehensive global data that reflects national implementation efforts in addition to and complementary to TrACSS [9]. While central government reporting used by TrACSS seems straightforward, there are currently no mechanisms to validate the accuracy of the reports submitted by countries. We hypothesized that public health experts would assess country efforts more critically than government officials (self-assessing their work) when completing the TrACSS. In complementing WHO coordinated TrACSS and published NAPs, we therefore intended to generate additional evidence through the perspective of national-level AMR experts. The main objectives of this study were to 1) to assess antimicrobial stewardship and national policies aimed at reducing antibiotic overuse or misuse in LMICs from an expert perspective 2) address potential data quality concerns regarding current AMR national implementation measures in LMICs 3) expand the range of data available from LMICs. For this goal, we launched a new global AMR survey in 2021, explicitly asking public health experts from countries to report on current efforts to address AMR in their respective countries. Our study does not include the examination of AMR within the domains of animal and environmental health.

## Methods

### Study design

This is a descriptive, cross-sectional study exploring data from a newly developed AMR survey tilted “Global Survey of Experts on Antimicrobial Resistance (GSEAR)”, conducted among public health experts in LMICs to assess the current policies and interventions in place through the expert perspectives.

### Study population

We used the World Bank's income classifications for 2019, which categorizes countries into Low Income, Lower Middle Income, Upper Middle Income, and High Income; we referred to the combined Low, Lower Middle, and Upper Middle Income categories as "low- and middle-income countries" (LMICs) [30]. We targeted all LMICs as classified by the World Bank in June 2019: 60 upper-middle, 47 lower-middle, and 31 low-income countries, yielding a total of 138 targeted LMICs [30]. In order to obtain the most accurate information possible, we constructed a novel database of public health AMR experts from all LMICs.

We followed a multi-step process to identify suitable country experts and used scientific output as a proxy for expertise. In a first step, we identified the ten top publishing public health researchers (which we will refer to as “experts” hereafter) in each country using the Web of Science publication database. We started this search by looking for publications in the broad areas of public health, health policy and services, and infectious diseases (Supplementary Table ST1 for search terms and syntax). If more than 200 publications were found for a country, we restricted specifically to focus on AMR using the terms provided in ST1. We then selected the ten authors with the highest number of relevant publications for our initial possible expert list. Email addresses for the experts in this initial list were obtained from publications and supplemented with google searches to identify author contact information. If no email address could be found online or the emails were no longer valid (bounce-back), these participants were replaced with the next highest ranked authors on the publication list. All experts who responded to the invitation to participate were asked to nominate other AMR experts who would be able to provide information on AMR policies and practices in their countries. These nominated experts were also invited to complete the survey.

### Study tools

We developed the GSEAR with the specific objective to evaluate components of the Global Action Plan on AMR [3]. The main areas covered by the survey in human health were: current antibiotic use (personal experience as well as their perception of general practices in their countries); antibiotic prescription practices; policies and interventions to control/restrict sale and consumption of antibiotics; existence of NAPs; collection, use, and reporting of surveillance data; and their experience of AMR awareness and community mobilization activities in their countries. The survey tool also included a section on personal background as well as a brief assessment of clinical antimicrobial and AMR knowledge, which we used to create a respondent specific AMR knowledge score. The ten questions used in the AMR knowledge score are intended to give a rough estimate of participants' knowledge on AMR. The survey also includes self-assessment questions to measure participants' familiarity with AMR and their experience in the field of AMR and public health. The survey instrument was pilot-tested in December 2020, during which we invited ten experts in five countries: Turkey, Tanzania, Bangladesh, Brazil, and Ethiopia to participate. The final survey tool is provided in Supplementary Table ST2.

### Data collection

All data was collected via an online survey to respondents, using the Open Data Kit (ODK) software platform, allowing them to complete the survey in English, French and Spanish. Invitations were sent to possible respondents with unique country-specific links via email. The initial email invitation was sent to all experts in the first quarter of 2021. Informed consent (Supplementary Table ST3) was obtained at the beginning of the online survey. The survey took approximately 15-20 minutes to complete. Participation was voluntary and no compensation was offered. Up to two reminders were sent if no response was received, at two and four weeks after the initial email. If no response was received after the second reminder, no additional contact attempts were made. Data collection was completed in May 2021.

### Ethical clearance

This study was reviewed and approved by Ethics Committee Northwest and Central Switzerland (EKNZ) according to HRA Art.51 (Statement ID: AO_2020_00026).

### Response scoring and country level aggregation

Responses were aggregated at the country-level. "Don't know/ Not sure" responses were not considered in determining country scores. Even though respondents were instructed to only answer questions if they were confident in the validity of their answers, responses to specific questions diverged in some instances. For national-level aggregated results, we generated a country-level median score as a first step. In the case of an equal number of divergent responses to an item within a country, we took the response from the respondents with higher antimicrobial knowledge scores as a tie-breaker and used their response. In the case of equal knowledge scores, we used the response of the experts with a longer residency in the country. In all cases, a clear modal response could be identified.

### Data analysis

We first created a flow chart to summarize participant identification and response patterns, ensuring transparency in the overall survey data collection process. In a second step, we compared the results of our expert survey to the results of the recent TrACSS 2020-2021 survey [20]. For this comparison, we identified four variables that were covered in both surveys: 1) the publicly-accessible existence (expert’s knowledge) of a NAP on AMR; 2) the presence of legislative policies restricting antimicrobial access (based on all survey questions regarding antimicrobial policies, coded as "AT LEAST ONE policy" to indicate their presence); 3) the current implementation of education/training programs for health professionals to promote rational use of antimicrobials; and 4) the existence of a national monitoring system for consumption and rational use of antimicrobials. A detailed description of the variables as well as the exact questions used to evaluate these four content areas is provided in Supplementary Tables ST4. Given that government officials may be reluctant to report lacking AMR efforts, our hypothesis was that public health experts would assess country efforts more critically than government officials typically completing the TrACSS survey, and thus on average report lower coverage of key programs to address AMR.

We then present newly collected data on key AMR variables that are not currently covered in the TrACSS, ranging from specific policies designed to reduce the risk of AMR to the actual implementation and enforcement of policies (Supplementary Table ST5). We also present data on quality assurance of antimicrobials and patient safety and policies within a country. Specifically, we present data on whether the country had i) legislation that required a prescription to get an antibiotic; ii) policies to reduce over-prescription of antibiotics in general by healthcare workers; iii) policies to limit healthcare worker prescription of protected or reserve antibiotics; iv) policies to restrict the over-the-counter sale of protected or reserve antibiotics. In addition, we evaluated whether national legislation of prescription requirements was enforced in practice in the countries; we assessed experts’ perception of the governments’ general interest and efforts in the area of AMR; and we estimated the proportion of countries where antibiotics could be obtained without prescription at pharmacies, drug shops or informal outlets. Lastly, we assessed the experts’ perception of potential availability of counterfeit antimicrobials in local markets.

## Results

**Fig. 1:**
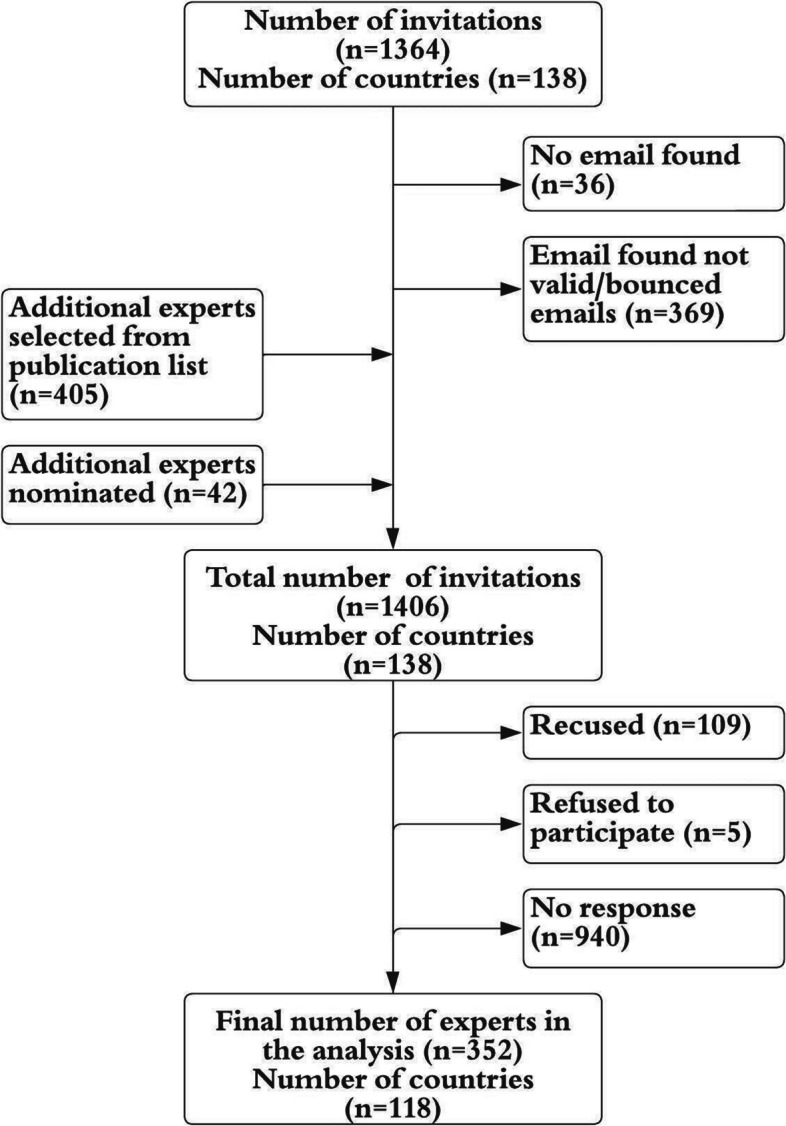
Flow diagram for survey data collection Figure 1 summarizes the expert recruitment and survey data collection process, including the survey turnout

Figure 1 summarizes the expert recruitment process as well as the survey participation. The web search yielded a total of 1364 public health experts across 138 countries. The targeted 10 experts were identified for 134 out of the 138 countries; a smaller number of experts could be identified for the Democratic People's Republic of Korea (*n*=6), Kyrgyz Republic (*n*=8), Turkmenistan (*n*=5) and Tuvalu (*n*=5). From this initial list, 405 experts were not reachable due to lacking or invalid email addresses and were replaced by the next-most eligible experts in their countries. An additional 42 experts were nominated by the experts first identified in the web search, resulting in a total of 1406 experts from 138 LMICs that were invited to complete the survey. Five respondents actively refused to participate, 109 recused themselves due to lacking familiarity with AMR policies in the country of interest, and 940 never responded to the invitation, resulting in a final sample 352 experts from 118 LMICs. 352 surveys were completed, for an overall response rate (including refusals and recusals) of 33% and survey completion rate (valid data obtained) of 25%.

**Fig. 2:**
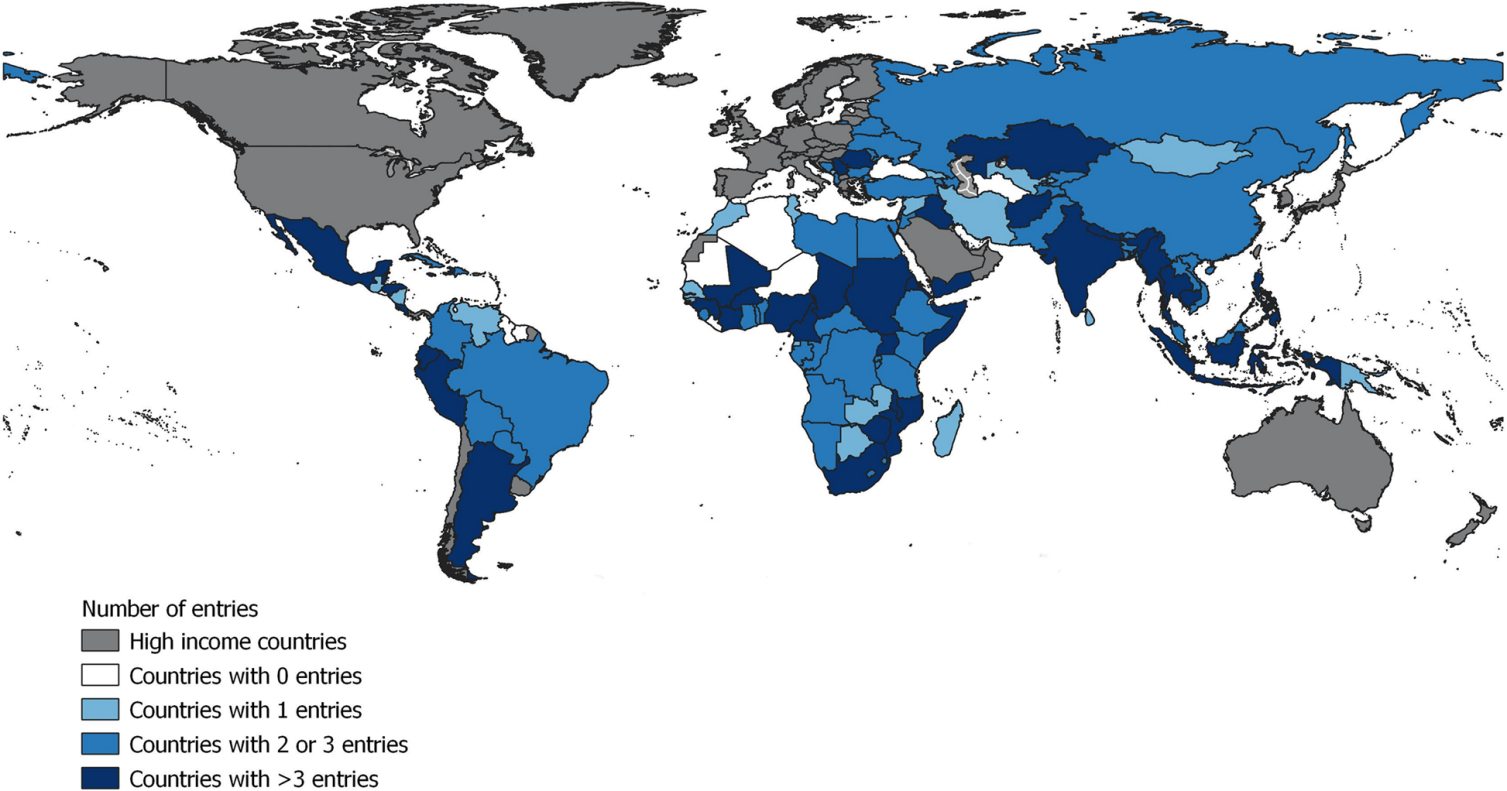
Number of completed surveys by country Notes: Figure 2 illustrates the geographical coverage of the survey and the number of completed responses per country, among the 118 LMICs. Countries omitted based on World Bank income classification are highlighted in grey

Figure 2 illustrates the geographical survey coverage and also shows the number of survey responses for each of the 118 LMICs in the final dataset. The average number of respondents obtained from each country was three; the country with the highest number of respondents was Nigeria (*n*=19). A full list of countries covered and number of responders per country is provided in Supplementary Table ST 6.

In the survey, most survey experts (97%) were between 25 and 64 years of age and 61% of the experts identified as male. Most of experts in the survey were identified as public health experts with clinical experience. Additionally, 69% of survey experts reported being currently active in clinical service. 76% of experts surveyed reported that they are actively working in the field of AMR and infectious diseases. 51% of experts had more than ten years of experience in public health, 20% had between five and ten years, and 15% had between one and five years, and the remainder (13%) had less than one year of experience (Table 1). Over three-quarters of the experts (82%) completed the survey in English, 9% completed it in French and 9% in Spanish. 74% of the experts were living in LMICs at the time of survey data collection. Only 26% of the respondents declared that they did not reside in the LMIC of interest for the last ten years.

**Table 1:** Characteristics of the experts in the survey

Characteristics	Freq (n)	Percent
Country Income classification		
Upper middle income	132	38%
Lower middle income	132	38%
Low income	88	25%
Region		
Sub-Saharan Africa	147	42%
Europe & Central Asia	56	16%
Latin America & Caribbean	56	16%
East Asia & Pacific	44	13%
Middle East & North Africa	25	7%
South Asia	24	7%
Gender		
Male	214	61%
Female	136	39%
Years of Age		
25-34	57	16%
35-44	111	32%
45-54	114	32%
55-64	57	16%
65+	12	3%
Clinical service		
Yes	238	69%
No	109	31%
Main field area of study		
Multidisciplinary including AMR	157	45%
AMR	61	17%
Infectious Diseases	48	14%
Others	36	10%
Public, Environmental & Occupational Health	26	7%
Health Policy & Services	24	7%
Type of institution and affiliation		
Academic Institution	178	51%
Mixed	75	21%
Public/government sectors	65	18%
NGOs, INGOs	22	6%
Private	12	3%
Years of experience in the field of AMR and public health		
Less than 1 year	46	13%
Within 1 to 5 years	54	15%
Within 5 to 10 years	71	20%
More than 10 years	181	51%
Language		
English	286	82%
French	33	9%
Spanish	33	9%
Currently residing in LMIC of interest		
Yes	260	74%
No	92	26%

### Comparison: GSEAR vs. TrACSS

**Fig. 3:**
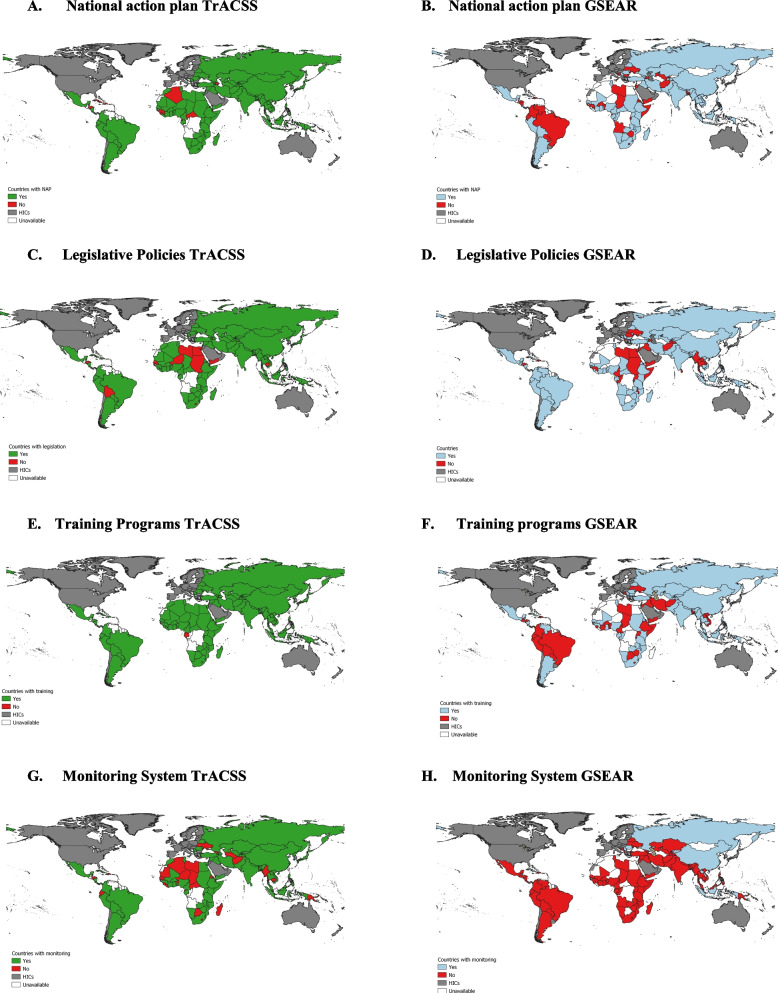
Key indicators of national AMR policies and practice evaluated TrACSS and GSEAR, **A** The publicly-accessible existence of a National Action Plan on AMR, TrACSS, **B** The publicly-accessible existence of a National Action Plan on AMR, GSEAR, **C** The presence of legislative policies restricting antibiotic use, TrACSS, **D** The presence of legislative policies restricting antimicrobial access, GSEAR, **E** National implementation of training programs for health professionals, TrACSS, **F** National implementation of training programs for health professionals, GSEAR, National monitoring system for use of antimicrobials, TrACSS National monitoring system for use of antimicrobials, GSEAR TrACSS: Green indicates countries with policies and interventions, red indicates countries without, grey indicates countries omitted based on World Bank income classification. White represents unavailable data. GSEAR: Countries with policies and interventions are shown in blue, those without in red, and those omitted due to World Bank income classification in grey. White represents unavailable data

The fifth round of TrACSS covered 163 countries globally, including 113 LMICs and was conducted in 2020-21. There were 16 LMICs covered in GSEAR but not in TrACSS, and 12 LMICs in TrACSS but not in the GSEAR. We found a substantial amount of disagreement between TrACSS and GSEAR in the four key measures captured in both surveys. As shown in Panel A of Fig. 3, TrACSS suggests almost universal coverage of NAPs (86%, Panel A), while GSEAR suggests that only two-third (67%, Panel B) of countries have a NAP to address AMR. We identified a similar pattern for legislative policies restricting antimicrobial access, where more gaps were identified in GSEAR compared to TrACSS (TrACSS 86%, Panel C: GSEAR 64%, Panel D). For national training programs for health professionals (Panel E&F), responses also diverged substantially across the two surveys: while 96% of countries have such programs according to TrACSS, according to GSEAR only 58% percent of countries have training programs for health professionals. Differences in findings across the two surveys were largest for the measure of availability of a national monitoring system for antimicrobial use (Panel G&H): while only 33% of countries (primarily in North Africa) do not have such systems according to TrACSS, according to GSEAR 90% of countries do not have such systems. The comparison between findings from TrACSS and GSEAR can be seen in supplementary figure SF1&2.

**Fig. 4:**
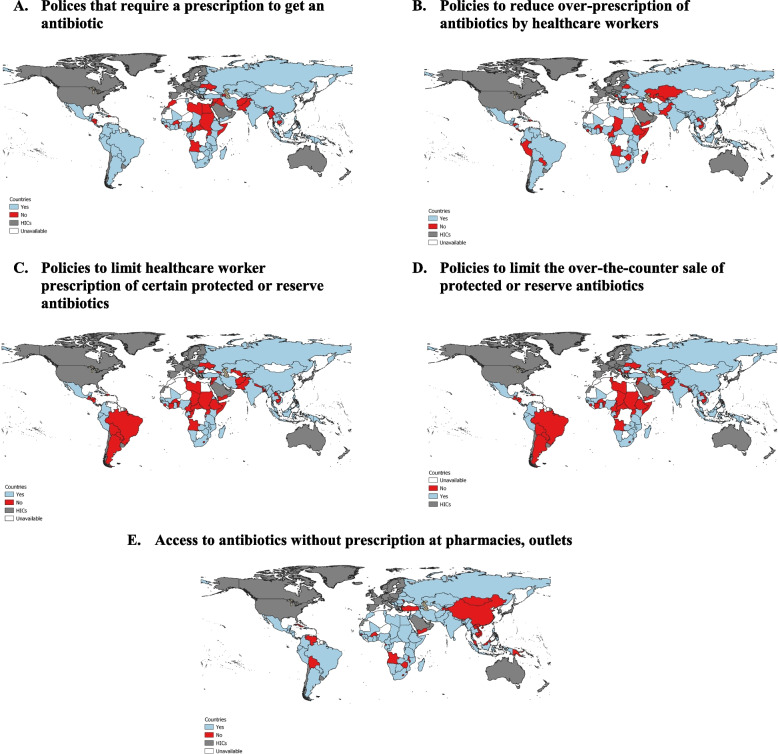
Prevalence of policies to restrict antibiotic use in LMICs according to GSEAR survey of experts GSEAR: Countries with policies and interventions are shown in blue, those without in red, and those omitted due to World Bank income classification in grey. White represents unavailable data

Figure 4 summarizes additional results regarding specific national policies on antibiotic use collected only in the GSEAR survey. According to experts, almost three quarters of countries (72%, Panel A) currently have policies requiring a prescription to get an antibiotic and 64% of LMICs (Panel B) have implemented policies to reduce the over-prescription of antibiotics by healthcare workers. As illustrated in Panel C, approximately half (51%) of countries have policies restricting healthcare worker prescriptions of specific protected or reserve antibiotics. The presence of specific policies regulating the over-the-counter sale of protected or reserve antibiotics were reported by 51% of countries (51%, Panel D). According to experts, policies that specifically restrict the use of protected or reserve antibiotics appear most lacking in Latin America and Sub-Saharan Africa (Fig 4, Panels C for healthcare workers and D for over the counter sales). Experts reported that getting an antibiotic without a prescription in practice was possible in almost three quarters of countries (74%, Panel E). There were only 30 countries where purchasing drugs without prescription was reported to be impossible.

**Fig. 5:**
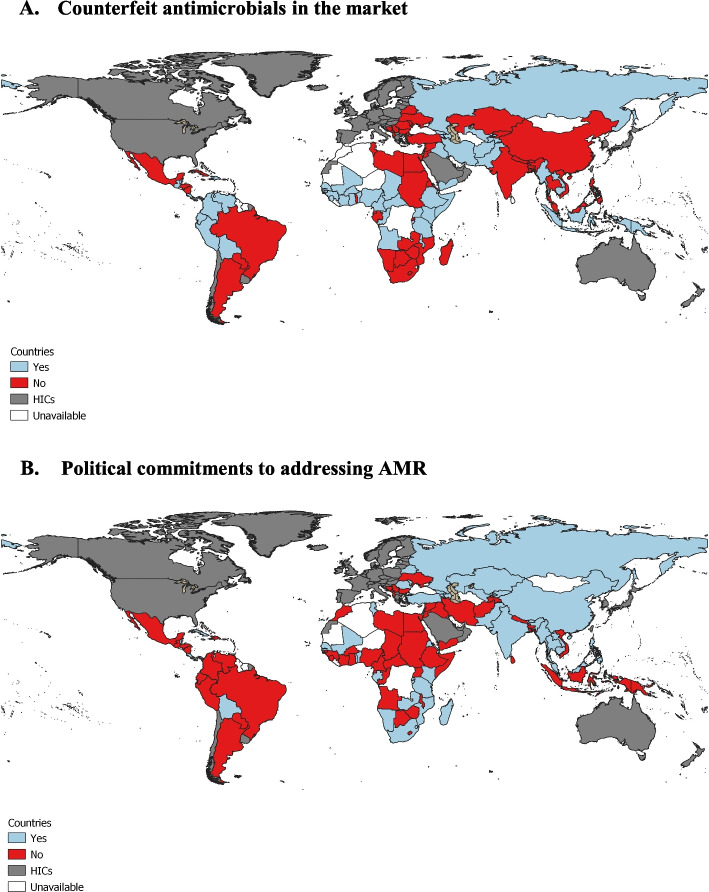
**A** Report of availability of counterfeit antimicrobials in the market, **B** Political commitment to addressing AMR from GSEAR survey GSEAR: Countries with policies and interventions are shown in blue, those without in red, and those omitted due to World Bank income classification in grey. White represents unavailable data

As shown in Fig. 5, Panel A, the presence of counterfeit or substandard antibiotics were reported by experts in 47% of countries, across the regions of South Africa, North Africa, the Middle East, East Asia, and Latin America. Government's current efforts to reduce AMR were reported to be present in 42% of countries (Fig. 5, Panel B), mostly Asian countries. The weakest political commitment to reduce the risk of AMR was reported by experts from countries in the Latin America and African regions.

## Discussion

The main objective of this study was to elicit experts’ perspectives on current coverage and implementation of policies to optimise antimicrobial use and active government efforts to slow the spread of AMR in LMICs. To our knowledge, this is the first global survey of public health experts aiming to directly assess current efforts of LMICs in implementing national response activities to optimise antimicrobial consumption – beyond the WHO coordinated TrACSS survey. This GSEAR collected information on a range of key measures related to national AMR policies on antimicrobial access and control, as reported by public health experts in AMR in 118 LMICs. Even though participation was slightly lower than what we had initially forecast, a total of 352 experts from 118 LMICs participated in this survey, for a final response rate of 33%. We used scientific output as a proxy of expertise in this area and most experts identified are independent public health professionals with clinical experience. Furthermore, a significant proportion of these experts are currently active in clinical service and working in the field of AMR and infectious diseases.

Additional findings from GSEAR not assessed in other surveys highlight specific national policies on antibiotic use, including prescription requirements, restrictions on prescribing by health professionals, and regulations on over-the-counter sales of protected antibiotics. Experts reported widespread availability of antibiotics without a prescription in practice, with variations between regions. Concerns about counterfeit or substandard antibiotics were reported in almost half of countries, with varying levels of government commitment to tackling AMR. These findings underscore the complexity and variability of AMR policies and practices around the world, and highlight the need for comprehensive and coordinated efforts to address this pressing public health issue.

There is substantial disagreement regarding the extent of policy implementation between TrACSS and GSEAR. Based on expert perspectives, there are significant gaps in current policy and implementation efforts to address AMR in LMICs, with a large number of countries falling short of target achievements; current policy coverage may be substantially lower than what the TrACSS survey suggests. The difference in results may be attributed to the nature of self-assessment surveys at the policy-making level, which tend to report more policy coverage and implementation work than when reported by experts. While reporting through central governments makes sense from an institutional perspective, it is important to note that in most cases, self-assessments by countries tend to over-report their policies and progress, which are not always aligned with experts' reports [31–33]. This highlights the need to consider the potential biases in self-assessment reports and the importance of obtaining accurate data. More robust data on what policies are in place, and the associated implementation and enforcement activities are also needed to track progress and ensure government accountability in addressing AMR. To determine the true extent to which governments are meeting their commitments in implementing AMR policies, it is essential to triangulate these individual perspectives, including those from TrACSS, with additional country-level research and impartial scientific assessments.

While it seems plausible that the experts consulted in the GSEAR survey may not always be aware of NAPs and specific policies or programs and government efforts available, the systematic gaps between TrACSS and GSEAR suggest that countries tend to over-report their efforts. Even if the data in TrACSS was accurate, the fact that local AMR experts are not aware of the existence of policies and programs suggests that the reach and impact of national measures – even if they exist – is likely limited. Systematic literature reviews, coupled with the establishment of a searchable database on AMR policies and interventions in LMICs, could potentially serve as solutions to triangulate and bridge the gap between expert perspectives and the information provided by countries in their self-assessments [34].

Overuse and misuse of antibiotics are the primary drivers of AMR [6, 35, 36]. The results presented here suggest that many relatively straightforward policies to reduce overuse and restrict inappropriate access to antibiotics are not currently in place in several LMICs. Expert findings suggest that the large gaps in policy and practice related to addressing AMR in LMICs, with a large number of countries failing to implement or substantiate agreed-on policy objectives with relevant enforcement or implementation. The results presented here also suggest major discrepancies between policies and actual implementation of these policies, particularly in the areas of over-the-counter antibiotic sales and monitoring of provider antibiotic prescribing behaviour. It is crucial to prioritize behaviour change programs in order to effectively educate healthcare providers in both hospital and community settings about the negative consequences of AMR and raise awareness among patients and the general public [37, 38].

In many low- and middle-income settings, over-the-counter antibiotic sales and counterfeiting persist, indicating a notable issue [7, 23, 39]. Previous evidence suggests that limited access to alternative healthcare options and effective disease prevention contributes to this problem, revealing broader structural inequities [40, 41]. Additionally, in low-income countries, the lack of antibiotic access poses a health threat of equal importance to AMR [42]. Considering noncompliance findings from our survey, there is a necessity to re-evaluate existing plans for practicality or make adjustments to the current governance framework to effectively address these structural constraints.

One of the five objectives of the Global Action Plan on AMR is to reduce unnecessary antibiotic prescriptions [3]. GSEAR indicates that several countries are not implementing certain policies associated with better antibiotic use, particularly those aimed at reducing over-prescription of antibiotics by healthcare workers. Although many countries require prescriptions, oversight, enforcement of the laws and policies appear to be lacking in many contexts [36]. Unregulated access to and sale of antibiotics in pharmacy outlets and among healthcare professionals were common in South Africa, North Africa, the Middle East, East Asia, and Latin America. Poorly regulated drug markets may also have resulted in widespread availability of substandard or counterfeit antibiotics over the counter and in local markets more generally, as reported by respondents [27]. Improved systems to control quality of available antibiotics and to regulate prescription to control innapropriate access are urgently needed [43, 44].

Even though the importance of surveillance on access and use was highlighted in the Global Action Plan on AMR and in the related literature, surveillance systems and access to and use of data from surveillance remains limited in many settings [3, 14, 36, 45, 46]. This aligns with the results of a recent study assessing the country's response to AMR in 114 LMICs [18, 19]. This may require improving laboratory diagnostic capacity in many settings [47]. Improved national monitoring systems are needed to guide countries on risk in their settings and to inform targeted approaches that address the specific needs and risks in their contexts. Policies and programs are required to improve appropriate access and use of antimicrobials, and specifically to control and limit inappropriate access to the use of reserve antibiotics. Efforts to address AMR should include coordinating with multiple sectors, including food production and safety, and economy particularly between human and animal health [3, 48].

### Study limitation

The study has several limitations. First, the methodology for identifying experts relies on academic citation metrics, which may not fully reflect scientific or societal impact. Resource constraints in LMICs, including publication barriers and difficulties in accessing proprietary databases, pose challenges. Relying on citation numbers alone in the selection of experts may also introduce bias, favouring researchers with privileged positions or access to international funding. Second, not all LMICs were covered by GSEAR, and some had few experts contributing, which may have affected the study's comprehensiveness. Additionally, the study's results may have been influenced by the expert selection process with a high proportion of non-responders or refusals among invited country experts. The significant disagreement between experts and central government indicates the subjectiveness of reporting based on experience perceptions. Survey questions can be interpreted differently by respondents, potentially leading to the misclassification of a country's policy status. Third, certain policies identified in previous studies, such as the existence of a Ministry of Health department dedicated to promoting the rational use of medicines (known to be associated with more appropriate antibiotic use), were not measured in our study. Additionally, the training variables used for comparison did not take into account the length of training, and any type of training or education program was considered without differentiation, which could also be a weakness. Lastly, the study did not consider key policy questions in the fields of animal and environmental health. Future studies should address these limitations. Despite these limitations, we believe this to be the first attempt at exploring expert insights. Our results would be nicely complemented with larger and more extensive future surveys. Nevertheless, we believe that the study results provide a novel and valuable insight into country policies and practices on antimicrobial stewardship and government action in the fight against AMR.

## Conclusion

Despite the presence of legislative policies to address AMR in many LMICs, the implementation and enforcement of these policies as well as surveillance systems to inform national practices and AMR risk remain limited. Increased national efforts, particularly in the areas of policy enforcement and improved monitoring of practices are urgently needed to reduce inappropriate antibiotic use and the further spread of AMR. Verified policy tracking and more high quality data on actual practices are also needed to track efforts and progress, and ensure both accountability and appropriate national responses. New interventions going beyond simply restricting antibiotic use in the local community are needed and should explicitly address the structural barriers and inequities driving behaviour in many settings.

### Supplementary Information

Supplementary Material 1.

## Data Availability

All data and figures relevant to this survey can be seen in the manuscript or supplementary materials. Access to the data can be obtained by emailing the corresponding author of the paper at guenther.fink@unibas.ch.
