# Supplementary material for: Coverage of policies to improve antimicrobial stewardship in human medicine in low and middle income countries: results from the Global Survey of Experts on Antimicrobial Resistance
Source: BMC Public Health. 2024 Aug 23;24:2297. doi: 10.1186/s12889-024-19542-2 (PMC11342495; doi:10.1186/s12889-024-19542-2)
Supplement: Supplementary file 1 — Supplementary Material 1. [file 12889_2024_19542_MOESM1_ESM.docx]

**Supplementary material**

**Table 1(ST1): Search term and syntax for the experts**

| Country name (CU) | Name of selected LMICs (eg. CU=Country name (e.g. CU=Angola or CU=Brazil) |
| --- | --- |
| Web of Science (WOS) categories | "Health Policy & Services" OR "Public, Environmental & Occupational Health" OR "Infectious Diseases" |
| Topic term (TS) for more than 200 publications | "Antimicrobial resistance" OR "Antibiotic resistance" OR "Antibiotics" OR "Microbial drug resistance" OR "Antimicrobial Drug Resistance" OR "Multiple drug resistance" |

**Table 2(ST2): Global Survey of Experts on Antimicrobial Resistance (GSEAR) survey tool**

| Introduction  1. Please choose your preferred language for this survey:  • English  • French  • Spanish  Consent  1.1 Are you willing to participate in this survey?  I have been well informed about the purpose of project and online survey.  I completely understand the possible advantages and disadvantages of involving in this survey.  I have been invited to participate and I accept to participate in this interview.  I allow my data and responses from this study to be used for medical research.  I consent voluntarily to be a participant in this study and understand that I have the rights to withdraw from the survey at any time without in any harm.  • Yes  • No  1.2 Allow data for further research purposes  • Yes  • No  Background information  2. Based on our publication record, we noticed you have worked extensively on ((Country)). Are you currently living in ((Country))?  • Yes  • No  2.1 Within the past 10 years, how many years did you live in ((Country))?  • Less than one year  • Between 1 to 2 years  • Between 3 to 5 years  • Between 5 to 10 years  • More than 10 years  • Not applicable  3. Do you have a current academic affiliation in ((Country))?  • Yes  • No  3.1 Please state the name of your academic or research institution.  4. In your own assessment, how familiar are you with the topic of antibiotic use and antimicrobial resistance in ((Country))?  • Not very familiar  • Familiar  • Very familiar  Questions related to experienced antibiotic use  For the following questions, please kindly choose “Don’t know/Not sure” if you are not sure about the answer.  We understand some of these questions may be difficult, so don't feel obliged to give an answer if you are not sure.  5. Are there any polices that require a prescription to get an antibiotic in ((Country))? (Select “Don’t know” if you are not sure).  • Yes  • No  • Don't know/ Not sure  6.1 Can a doctor/ general practitioner prescribe antibiotics in ((Country))?  • Yes  • No  • Don't know/ Not sure  6.2 Can a dentist prescribe antibiotics in ((Country))?  • Yes  • No  • Don't know/ Not sure  6.3 Can a mid-level provider such as an assistant medical officer (AMO) or clinical officer (CO) prescribe antibiotics in ((Country))?  • Yes  • No  • Don't know/ Not sure  6.4 Can a nurse prescribe antibiotics in ((Country))?  • Yes  • No  • Don't know/ Not sure  6.5 Can a pharmacist prescribe antibiotics in ((Country))?  • Yes  • No  • Don't know/ Not sure  6.6 Can a trained community health worker/ volunteer health worker prescribe antibiotics in ((Country))?  • Yes  • No  • Don't know/ Not sure  6.7 Are there any other professionals who can prescribe antibiotics in ((Country))?  • Yes  • No  • Don't know/ Not sure  6.8 Please specify the professionals who can prescribe antibiotics in ((Country)).  Questions related to experienced antibiotic use  (Select “Don’t know” if you are not sure).  7. Are you aware of any regulatory and legislative policies to ban the over-the counter sale of antibiotics without prescription in ((Country))?  • Yes, the country has policies to ban the sale of over-the counter antibiotics.  • No, the country does not have policies to ban the sale of over-the counter antibiotics.  • Don't know/ Not sure  8. Are there any policies to limit health care worker prescription of certain protected or reserve antibiotics in ((Country))?  • Yes  • No  • Don't know/ Not sure  9. Are there any policies to limit the over-the-counter sale of protected or reserve antibiotics in ((Country))?  • Yes  • No  • Don't know/ Not sure  10. Based on your experience, how common is antibiotic self-medication (the use of antibiotics without prescription) in your country?  • Very Common  • Common  • Neither common nor uncommon  • Uncommon  • Very uncommon  • Don't know/ Not sure  Questions related to antibiotic prescription  Now I would like to ask you a few questions about how easy it is to get antibiotics without prescription in ((Country)). Could you tell me whether it is possible to get…?  (Select “Don’t know” if you are not sure).  11.1 ...antibiotics without prescription in a medical store or pharmacy (with a trained pharmacist) in (Country))?  • Yes  • No  • Don't know/Not sure  11.2 …antibiotics without prescription in drug shops in ((Country))?  • Yes  • No  • Don't know/Not sure  11.3 ...antibiotics without prescription from local vendors/ informal outlets/ patent medical store in ((Country))?  • Yes  • No  • Don't know/Not sure  11.4 ...get antibiotics without prescription in supermarkets or groceries in ((Country))?  • Yes  • No  • Don't know/Not sure  10.5 ...antibiotics without prescription in online pharmacies/ from the internet in ((Country))?  • Yes  • No  • Don't know/Not sure  10.6 ...get antibiotics without prescription from traditional healers in ((Country))?  • Yes  • No  • Don't know/Not sure  10.7 ...get antibiotics without prescription from any other place or sources in ((Country))?  • Yes  • No  • Don't know/Not sure  10.8 Please name any other places or sources to get antibiotics without prescription in ((Country))?  Questions related to antibiotic prescription  11. One of the challenges faced by many countries is to find a balance between equitable access to antibiotics and avoiding excess use. How would you assess overall antibiotics access and use in ((Country))?  • Highly Insufficient  • Somewhat insufficient  • About the right amount/Appropriate  • A little too much  • Way too much  • Don't know/ Not sure  12. How often does it happen that an antibiotic prescribed by a doctor or nurse cannot be obtained/accessed by a patient in ((Country))?  • Never  • Rarely  • Sometimes  • Often  • Always  • Somewhat unknown/Don't know  13. How often are health workers forced to prescribe antibiotics that are not their first choice for the patient because the ideal antibiotic is not available?  • Never  • Rarely  • Sometimes  • Often  • Always  • Somewhat unknown/Don't know  14. Have you heard of unregistered or illegal local production of antibiotics in ((Country))?  • Yes  • No  • Don't Know/ Not sure  15. Have you heard of illegal imports of antibiotics in ((Country))?  • Yes  • No  • Don't Know/ Not sure  16. Have you ever seen or heard of the availability of counterfeit antibiotic drugs in the market?  • Yes  • No  • Don't Know/ Not sure  Questions related to AMR policies and interventions  17.1 Does ((Country)) have national treatment guidelines to improve antibiotic prescribing practices?  • Yes  • No  • Don't Know/ Not sure  17.2 Does ((Country)) have policies to reduce over-prescription of antibiotics by health workers?  • Yes  • No  • Don't Know/ Not sure  17.3 Does ((Country)) have mass media campaign to control self-medication (the use of antibiotics without prescription)?  • Yes  • No  • Don't Know/ Not sure  17.4 Does ((Country)) have financial incentives or pay for performance programs for health care providers or for health facilities to improve antibiotic prescribing practices?  • Yes  • No  • Don't Know/ Not sure  17.5 Does ((Country)) have education programs for health professionals to promote rational use of antibiotics?  • Yes  • No  • Don't Know/ Not sure  Questions related to AMR policies and interventions  18. Does ((Country)) have policies that limit antibiotic prescribing or prescription of certain reserve or protected antimicrobials to medical providers with special training or certification in infectious disease?  • Yes  • No  • Don't Know/ Not sure  19. Are antibiotic prescription rates monitored at the health care provider level in ((Country))?  • Yes  • No  • Don't Know/ Not sure  19.1 What institution or authority monitors antibiotic prescription rates at the health care provider level in ((Country))?  19.2 What are the consequences of high prescription rates by health care providers?  20. Are antibiotic prescription rates monitored at the health facility level in ((Country))?  • Yes  • No  • Don't Know/ Not sure  20.1 What institution or authority in ((Country)) monitors antibiotic prescription rates at the health facility level in ((Country))?  20.2 What are the consequences of high prescription rates by health facilities?  Questions related to AMR policies and interventions  21. Are electronic clinical decision support algorithms or tools for health care workers used to guide antimicrobial prescribing decisions in ((Country))?  • Yes  • No  • Don't Know/ Not sure  22. Is influenza testing available in hospitals or health facilities in ((Country)) to distinguish viral from bacterial infections?  • Such tests are not available in hospitals or health facilities to my knowledge  • Such tests are available in only a few selected hospitals or health facilities  • Such tests are available in more than half of the hospitals or health facilities  • Such tests are available in most hospitals or health facilities  • Don't Know/ Not sure  23. Is point of care C-reactive protein (CRP) used by health care workers in hospitals or health facilities in ((Country)) to guide decisions on the use of antimicrobial therapy?  • Such tests are not available in hospitals or health facilities to my knowledge  • Such tests are available in only a few selected hospitals or health facilities  • Such tests are available in more than half of the hospitals or health facilities  • Such tests are available in most hospitals or health facilities  • Don't Know/ Not sure  24. Is point of care rotavirus testing available in hospitals or health facilities in ((Country)) to guide decisions on the use of antimicrobial therapy?  • Such tests are not available in hospitals or health facilities to my knowledge  • Such tests are available in only a few selected hospitals or health facilities  • Such tests are available in more than half of the hospitals or health facilities  • Such tests are available in most hospitals or health facilities  • Don't Know/ Not sure  25. Are there any other policies or national programs to address rationale antibiotic prescriptions, sales and use in ((Country))?  • Yes  • No  • Don't know/Not sure  25.1 Please specify the policies or strategies to control antibiotic prescriptions, sales and use in ((Country)):  Questions related to AMR policies and interventions  26. Most countries state that political commitment is crucial for combatting the problem of antimicrobial resistance. Giving your experience, how would you assess the political commitment towards reducing antimicrobial resistance in ((Country))?  • Strong  • Somewhat strong  • Average  • Limited  • Somewhat limited  • Not at all  • Don't know/Not sure  27. What do you perceive as the biggest challenges or issues related to antimicrobial resistance in (Country))?  28. In your opinion, what policy or interventional approach is most needed for effectively addressing rational antibiotic use in ((Country))?  Questions related to AMR national action plan  29. Does the country have a national AMR action plan or guideline to combat the threat of resistance in ((Country))?  • Yes  • No  • Don't know/ Not sure  30. Is there a tracking and monitoring plan within the AMR that defines specific indicators for evaluating the implementation and outputs of the AMR action plan in ((Country))?  • Yes  • No  • Don't know/ Not sure  31. Have you ever been involved in the development process or provided technical inputs to a national AMR action plan or guideline in ((Country))?  • Yes  • No  • Can't remember  31.1 What sources of information, recommendations or guidelines were considered in development of the national action plan, if any?  • Global recommendations  • Guidelines or recommendations from neighbouring or countries in the region  • National surveillance data  • National expert opinion  • Other  • Don't know/ Not sure  32. Please specify the other sources of information, recommendations or guidelines used in development of the national action plan?  33. In your opinion, does the government of ((Country)) take research and new evidence into consideration for AMR policy making?  • Not at all  • Only marginally  • Sometimes  • Most of the time  • Always  • Don't know/ Not sure  34. What kind of research would be needed to improve AMR policies in ((Country))?  Questions related to surveillance data  35. Have you ever been involved in Antimicrobial Stewardship (AMS) programs to promote rational use of antimicrobials in ((Country))?  • Yes  • No  • Can't remember  36. Have you ever taken the role to report and update the results of the AMR surveillance activities to policy makers or other governing bodies in ((Country))?  • Yes  • No  • Can't remember  37. How are national AMR surveillance data and reports shared ((Country))?  • Widely distributed and publicized  • Available upon request by anyone  • Available only to researchers or professionals with a relevant role, job or field  • Available only to select key national stakeholders  • Not available  • Don't know  38. Have you ever been involved or joined collaborative work to combat the threat of AMR at regional and international level?  • Yes  • No  • Can't remember  Questions related to awareness  39. Have you ever received training, education, or participated in a capacity-building programme regarding rational antimicrobial use in healthcare settings in ((Country))?  • Yes  • No  • Can't remember  40. Have you ever trained others on rational antimicrobial use in healthcare settings in ((Country))?  • Yes  • No  • Can't remember  41. If you had to guess: what proportion of general population is aware of the problem of antimicrobial resistance in ((Country))?  • 100% of the general population aware of AMR problem  • 80% of the general population aware of AMR problem  • 60% of the general population aware of AMR problem  • 40% of the general population aware of AMR problem  • 20% of the general population aware of AMR problem  • 0% of the general population aware of AMR problem  • Don't know/ Not sure  Questions related to own recent exposure to antibiotics  42. Please confirm the country of your current residence: please select the country from the following list:  • Afghanistan  • Albania  • Algeria  • Andorra  • Angola  • Antigua and Barbuda  • Argentina  • Armenia  • Australia  • Austria  • Azerbaijan  • Bahamas  • Bahrain  • Bangladesh  • Barbados  • Belarus  • Belgium  • Belize  • Benin  • Bhutan  • Bolivia  • Bosnia and Herzegovina  • Botswana  • Brazil  • Brunei  • Bulgaria  • Burkina Faso  • Burundi  • Côte d'Ivoire  • Cabo Verde  • Cambodia  • Cameroon  • Canada  • Central African Republic  • Chad  • Chile  • China  • Colombia  • Comoros  • Congo (Congo-Brazzaville)  • Costa Rica  • Croatia  • Cuba  • Cyprus  • Czechia (Czech Republic)  • Democratic Republic of the Congo  • Denmark  • Djibouti  • Dominica  • Dominican Republic  • Ecuador  • Egypt  • El Salvador  • Equatorial Guinea  • Eritrea  • Estonia  • Eswatini (fmr. "Swaziland")  • ((Country))  • Fiji  • Finland  • France  • Gabon  • Gambia  • Georgia  • Germany  • Ghana  • Greece  • Grenada  • Guatemala  • Guinea  • Guinea-Bissau  • Guyana  • Haiti  • Holy See  • Honduras  • Hungary  • Iceland  • India  • Indonesia  • Iran  • Iraq  • Ireland  • Israel  • Italy  • Jamaica  • Japan  • Jordan  • Kazakhstan  • Kenya  • Kiribati  • Kuwait  • Kyrgyzstan  • Laos  • Latvia  • Lebanon  • Lesotho  • Liberia  • Libya  • Liechtenstein  • Lithuania  • Luxembourg  • Madagascar  • Malawi  • Malaysia  • Maldives  • Mali  • Malta  • Marshall Islands  • Mauritania  • Mauritius  • Mexico  • Micronesia  • Moldova  • Monaco  • Mongolia  • Montenegro  • Morocco  • Mozambique  • Myanmar (formerly Burma)  • Namibia  • Nauru  • Nepal  • Netherlands  • New Zealand  • Nicaragua  • Niger  • Nigeria  • North Korea  • North Macedonia  • Norway  • Oman  • Pakistan  • Palau  • Palestine State  • Panama  • Papua New Guinea  • Paraguay  • Peru  • Philippines  • Poland  • Portugal  • Qatar  • Romania  • Russia  • Rwanda  • Saint Kitts and Nevis  • Saint Lucia  • Saint Vincent and the Grenadines  • Samoa  • San Marino  • Sao Tome and Principe  • Saudi Arabia  • Senegal  • Serbia  • Seychelles  • Sierra Leone  • Singapore  • Slovakia  • Slovenia  • Solomon Islands  • Somalia  • South Africa  • South Korea  • South Sudan  • Spain  • Sri Lanka  • Sudan  • Suriname  • Sweden  • Switzerland  • Syria  • Tajikistan  • Tanzania  • Thailand  • Timor-Leste  • Togo  • Tonga  • Trinidad and Tobago  • Tunisia  • Turkey  • Turkmenistan  • Tuvalu  • Uganda  • Ukraine  • United Arab Emirates  • United Kingdom  • United States of America  • Uruguay  • Uzbekistan  • Vanuatu  • Venezuela  • Vietnam  • Yemen  • Zambia  • Zimbabwe  43. Last time you were sick with a fever, did you take an antibiotic?  • Yes  • No  • Can't remember  43.1 If you took an antibiotic the last time you were sick with fever, when was this?  • Within the last 4 weeks  • Within the last six months  • More than six months and less than one year  • Within one to two years  • More than two years  • Can't remember  44. If you did not take an antibiotic for your last episode of illness with fever, when was the last time you took an antibiotic in your current country of residence?  • Within the last 4 weeks  • Within the last six months  • More than six months and less than one year  • Within one to two years  • More than two years  • Can't remember  45. Did you need a prescription the last time you took an antibiotic?  • Yes  • No  • Can't remember  46. Do you have children younger than five year of age who live with you?  • Yes  • No  46.1 Last time your child less than five years was sick with fever (if you have multiple children under age 5, please answer for the youngest one), did your child take an antibiotic?  • Yes  • No  • Can't remember  46.2 If your child took an antibiotic, when was it?  • Within the last four weeks  • Within the last six months  • More than six months and less than one year  • Within one to two years  • More than two years  • Can't remember  46.3 Did you need a prescription to get an antibiotic for your child’s last episode of illness?  • Yes  • No  • Can't remember  47. In your experience, how easy is it in general to get antibiotics without prescription at pharmacies, drugs shops or informal outlets in the country of your current residence?  • Very Easy  • Easy  • Somewhat easy  • Somewhat hard  • Impossible  • Don’t know/Not sure  Knowledge based question  48. Antimicrobial resistance (AMR) is defined as the capability of bacteria, parasites, viruses and fungi to grow and spread in the presence of antimicrobial medicine that are typically used to treat infections.  In your opinion, which of the following statements are True or False?  48.1 Drug resistant bacteria can spread from person to person.  • True  • False  • Don't know/Not sure  48.2 Antibiotics are effective against both bacterial and viral infections.  • True  • False  • Don't know/Not sure  48.3 The length of illness can be shortened in fever and flu cases by taking early treatment with antibiotics.  • True  • False  • Don't know/Not sure  48.4 Vaccination and immunization program have no major impact on the problem of antibiotic resistance.  • True  • False  • Don't know/Not sure  48.5 Clean water, hand hygiene and sanitation program can prevent infections and thereby reduce the need for antimicrobials, and slow the process of increased antimicrobial  resistance in a setting.  • True  • False  • Don't know/Not sure  Knowledge based question  49. For which of the following conditions do you consider the use of antibiotics appropriate? (Multiple choice: Response may be more than one)  • Common cold or flu  • Headaches  • Uncomplicated diarrhea (without blood in stool)  • Bacterial pneumonia  • Viral infection  50. Which of the following is/are penicillin derivatives or penicillin containing antibiotics? (Multiple choice: Response may be more than one)  • Benzylpenicillin  • Amoxicillin  • Ciprofloxacin  • Ampicillin  • Norfloxacin  • Tetracyclines  • Gentamicin  • Clarithromycin  • Clavulanic acid  51. What is the recommended course of amoxicillin treatment for mild to moderate bronchitis in an adult patient in your country?  • 500 mg orally every 8 hours or 875 mg every 12 hours for a minimum of 2 days  • 500 mg orally every 8 hours or 875 mg every 12 hours for a minimum of 14 days  • 500 mg orally every 8 hours or 875 mg every 12 hours for a minimum of 5 days  • 500 mg orally every 8 hours or 875 mg every 12 hours until symptoms relieve  • Don't know/Not sure  52. What is the recommended course of Amoxicillin-clavulanate for recurrent tonsillitis caused by Streptococcus pneumonia for a child in your country?  • 200 mg/5 mL or 400 mg/5 mL oral suspension or chewable tablets: 12.5 to 22.5 mg/kg orally every 12 hours for a minimum of 2 days  • 200 mg/5 mL or 400 mg/5 mL oral suspension or chewable tablets: 12.5 to 22.5 mg/kg orally every 12 hours for a minimum of 5-7 days  • 200 mg/5 mL or 400 mg/5 mL oral suspension or chewable tablets: 12.5 to 22.5 mg/kg orally every 12 hours for a minimum of 21 days  • 200 mg/5 mL or 400 mg/5 mL oral suspension or chewable tablets: 12.5 to 22.5 mg/kg orally every 12 hours until symptoms relieve  • Don't know/Not sure  53. When I am on antibiotic treatment, I contribute to the risk of developing drug resistance.  • Yes  • No  • Don't Know/ Not sure  General questions  54. Please choose gender identity.  • Male  • Female  • Don't want to mention  55. Please indicate your age group.  • 19-24  • 25-34  • 35-44  • 45-54  • 55-64  • 65+  56. Please indicate the type of your institution and affiliation. (Multiple choice: Response may be more than one)  • Public/government sectors  • Private  • Academic Institution  • NGOs, INGOs or religious institution  • Not known or unspecified  • Other  56.1 Other type of institution and affiliation. Please specify.  57. What is/are your field of training? (Multiple choice: Response may be more than one)  • Medicine  • Medical services  • Nursing  • Dental studies  • Public Health  • Veterinary  • Microbiologist  • Not known or unspecified  • Other  57.1 Other type of field training. Please specify.  58. Have you ever conducted clinical service in your professional career?  • Yes  • No  • Don't know  59. Which of following best describes your expertise and main field area of study?(Multiple choice: response may be more than one)  • Antimicrobial resistance  • Health Policy & Services  • Public, Environmental & Occupational Health  • Infectious Diseases  • Other  59.1 Other main field area of study. Please specify.  60. Please indicate total years of experience in the field of public health.  • Not at all  • Less than 1 year  • Within 1 to 5 years  • Within 5 to 10 years  • More than 10 years  61. Overall, how would you assess your knowledge of antibiotic use and antimicrobial resistance in [country] on a scale from 1 (very limited) to 10 (expert)?  62. Would you like to receive a copy of the final paper resulting from the survey?  • Yes  • No  63. I highly appreciate your time and input. Please kindly provide your email below so we  know you have completed this survey and can later share the results of this study with you.  This is the end of an online survey. Thank you for your time and participation.  Thank you for your time and consideration.  End of online survey |
| --- |

**Table 3 (ST3): Informed consent**

| Background and purpose  My name is Kyaw Zay Ya. I am a PhD student at the Swiss Tropical and Public Health Institute (Swiss TPH) interested in the global use of antibiotics and antimicrobial resistance (AMR). I am currently working on a study titled “Global implementation of antimicrobial resistance policies as well as their relationship to antibiotic use”. The aim of this study is to assess the extent to which antibiotic resistance prevention policies and interventions are currently being implemented in low and middle-income countries. Given that very little systematic information is currently available on antibiotic use in low and middle-income countries (LMICs), I hope to collect information on this topic through this online survey. I am contacting you today because I found several of your publications in this area, and as an expert in this topic area, I hope you will be able to help me with this study.    Informed consent  How will the study be conducted?  In order to include as many LMICs as possible, we identified public health experts and researchers in as many LMIC countries as possible. A structured online questionnaire regarding antibiotic use and antimicrobial resistance policies will be sent via email to everybody on this list, including yourself. This survey will take approximately 10-15 minutes of your time, if you choose to complete it.  What are the risks?  There will be not be any direct risk nor complications to the participants in the survey. The participants can skip questions, stop or quit completing the survey at any time during the survey. There will be no harm or penalty for failing to complete the survey.  Are there any advantages?  There will be no direct benefit from participating in this survey. Your participation is likely to help us to understand global use of antibiotics as well as the implementation of AMR policies globally.  Data and confidentiality  A unique participant code will be assigned to public health researchers who complete the survey. The information you provide will be kept strictly confidential and your name will not be included in any study report or publication. Only project members will have access to survey data.  Insurance, compensation  Participation in this survey is strictly voluntary. We will not be able to provide you with any compensation for completing this survey.  Voluntariness  Participation in this study is voluntary. Your contribution in this interview will be highly appreciated and acknowledged.  Study funding  This project has received funding from the European Union’s Horizon 2020 research and innovation programme under the Marie Skłodowska-Curie grant agreement No 801076, through the SSPH+ Global PhD Fellowship Programme in Public Health Sciences (GlobalP3HS) of the Swiss School of Public Health.  Responsibility  If you need more information about this study or wish to ask the questions later, please feel free to contact me by email: Dr.Kyaw Zay Ya, PhD fellow, Swiss Tropical and Public Health Institute email: kyaw.zayya@swisstph.ch or by phone at +41-0787844144. |
| --- |

**Table 4 (ST4): Variables comparison for GSEAR and TrACSS**

| GSEAR variable | TrACSS variable |
| --- | --- |
| plan: Survey NAP on AMR  Code: Yes, No, Don't know/ Not sure  Does the country have a national AMR action plan or guideline to combat the threat of resistance in ((Country))?  • Yes  • No  • Don't know/ Not sure | TrACSSplan: TrACSS survey NAP on AMR  Code: Yes for C & D & E, No for A & B  Country progress with development of a national action plan on AMR * A - No national AMR action plan. B - National AMR action plan under development. C - National AMR action plan developed. D - National AMR action plan being implemented. E - National AMR action plan being implemented and actively monitored through a monitoring and evaluation framework. |
| legi: survey legislative policies on antibiotic use  Code: Yes, No  This variable is the combination of all survey questions related to legislative policies on antibiotic use. Combine all of survey questions: one if any of the following is YES.  Are there any polices that require a prescription to get an antibiotic in ((Country))? (Select “Don’t know” if you are not sure).  • Yes  • No  • Don't know/ Not sure  Are you aware of any regulatory and legislative policies to ban the over-the counter ale of antibiotics without prescription in ((Country))?  • Yes, the country has policies to ban the sale of over-the counter antibiotics.  • No, the country does not have policies to ban the sale of over-the counter antibiotics.  • Don't know/ Not sure  Are there any policies to limit health care worker prescription of certain protected or reserve antibiotics in ((Country))?  • Yes  • No  • Don't know/ Not sure  Are there any policies to limit the over-the-counter sale of protected or reserve antibiotics in ((Country))?  • Yes  • No  • Don't know/ Not sure  Does ((Country)) have policies to reduce over-prescription of antibiotics by health workers?  • Yes  • No  • Don't Know/ Not sure | TrACSSlegi: TrACSS survey legislative policies on antibiotic use  Code: Yes, No, Don't know  Country legislations on antimicrobial use [Country has laws or regulations on prescription and sale of antimicrobials, for human use.] Yes No Don't know |
| train: survey training programs for health professionals  Code: Yes, No, Don't know/ Not sure  17.5 Does ((Country)) have education programs for health professionals to promote rational use of antibiotics?  • Yes  • No  • Don't Know/ Not sure | TrACSStrain: TrACSS survey training programs for health professionals  Code: No for A, Yes for B,C, D & E  6.2 Training and professional education on AMR in the human health sector * A - No training for human health workers on AMR. B - Ad hoc AMR training courses in some human health related disciplines. C - AMR is covered in 1) some pre-service training and in 2) some in-service training or other continuing professional development (CPD) for human health workers. D - AMR is covered in pre-service training for all relevant cadres. In-service training or other CPD covering AMR is available for all types of human health workers nationwide. E - AMR is systematically and formally incorporated in pre-service training curricula for all relevant human health cadres. Relevant groups for human health nationwide, in public and private sectors take up in-service training or other CPD on AMR. |
| monitor: survey national monitoring system  Code: Yes, No  This variable is the combination of all survey questions related to antibiotic prescription rate monitoring at health workers and facilities level: one if any of the question is YES.  Are antibiotic prescription rates monitored at the health care provider level in ((Country))? • Yes • No • Don't Know/ Not sure Are antibiotic prescription rates monitored at the health facility level in ((Country))? • Yes • No • Don't Know/ Not sure | TrACSSmonitor: TrACSS survey national monitoring system  Code: No for A, Yes for B,C, D & E  .1 National monitoring system for consumption and rational use of antimicrobials in human health  A - No national plan or system for monitoring use of antimicrobials.  B - System designed for surveillance of antimicrobial use that includes monitoring national level sales or consumption of antibiotics in health services.  C - Total sales of antimicrobials are monitored at national level and/or some monitoring of antibiotic use at sub-national level.  D - Prescribing practices and appropriate antibiotic use are monitored in a national sample of healthcare settings.  E - On a regular basis (every year/two years) data is collected and reported on: a) Antimicrobial sales or consumption at national level for human use; and b) Antibiotic prescribing and appropriate/rational use, in a representative sample of health facilities, public and private. |

**Table 5 (ST5): Key AMR variables covered by GSEAR and TrACSS**

| Level | Area | Key variables | GSEAR | TrACSS |
| --- | --- | --- | --- | --- |
| Policy making level | Antibiotic sale | Legislative policies on antibiotic use | **(+)** | **(+)** |
|  | Plan and Guidelines | National Action Plan on AMR | **(+)** | **(+)** |
|  | Political commitment | Political commitment on AMR | **(+)** |  |
| Implementation | Antibiotic prescription | Policies that require a prescription to get an antibiotic | **(+)** |  |
|  |  | Policies to reduce over-prescription of antibiotics by health workers | **(+)** |  |
|  | Protected or reserved antibiotics | Policies to limit health care worker prescription of certain protected or reserved antibiotics | **(+)** |  |
|  |  | policies to restrict the over-the-counter sale of certain protected or reserved antibiotics | **(+)** |  |
|  | Training | Training programs for health professionals | **(+)** | **(+)** |
|  | Monitoring | Monitoring system for consumption and rational use of antimicrobials | **(+)** | **(+)** |
|  | Quality assurance and patient safety | the availability of counterfeit antibiotic drugs in the country | **(+)** |  |
| End-user level | Access | How easy to get antibiotics without prescription at pharmacies, drug shops or informal outlets | **(+)** |  |

**Table 6 (ST6): Countries coverage by GSEAR**

Countries covered by TrACSS and GSEAR

| Country | Number of responses |
| --- | --- |
| Afghanistan | 5 |
| Albania | 2 |
| Angola | 2 |
| Argentina | 4 |
| Armenia | 2 |
| Azerbaijan | 2 |
| Bangladesh | 2 |
| Belarus | 2 |
| Belize | 4 |
| Benin | 2 |
| Bhutan | 2 |
| Bolivia | 2 |
| Bosnia and Herzegovina | 3 |
| Botswana | 1 |
| Brazil | 2 |
| Bulgaria | 2 |
| Burkina Faso | 4 |
| Burma/Myanmar | 7 |
| Burundi | 3 |
| Cambodia | 5 |
| Cameroon | 4 |
| Cape Verde | 1 |
| Central African Republic | 2 |
| Chad | 4 |
| China | 2 |
| Colombia | 2 |
| Comoros | 1 |
| Congo | 2 |
| Congo(Democratic Republic of the) | 5 |
| Costa Rica | 5 |
| Cuba | 2 |
| Dominican Republic | 3 |
| Ecuador | 5 |
| Egypt | 2 |
| Equatorial Guinea | 1 |
| Eritrea | 3 |
| Ethiopia | 2 |
| Gabon | 3 |
| Gambia | 2 |
| Gaza Strip | 3 |
| Georgia | 1 |
| Ghana | 3 |
| Grenada | 2 |
| Guatemala | 1 |
| Guinea | 6 |
| Guinea-Bissau | 1 |
| Haiti | 4 |
| Honduras | 4 |
| India | 7 |
| Indonesia | 4 |
| Iran | 1 |
| Iraq | 4 |
| Ivory Coast | 7 |
| Jamaica | 4 |
| Jordan | 3 |
| Kazakhstan | 5 |
| Kenya | 3 |
| Kiribati | 1 |
| Kosovo | 2 |
| Kyrgyzstan | 2 |
| Laos | 2 |
| Lebanon | 2 |
| Lesotho | 2 |
| Libya | 2 |
| Madagascar | 1 |
| Malawi | 5 |
| Malaysia | 3 |
| Maldives | 1 |
| Mali | 5 |
| Marshall Islands | 1 |
| Mauritius | 2 |
| Mexico | 4 |
| Moldova | 2 |
| Mongolia | 1 |
| Montenegro | 1 |
| Morocco | 1 |
| Mozambique | 4 |
| Namibia | 2 |
| Nepal | 3 |
| Nicaragua | 1 |
| Nigeria | 19 |
| North Macedonia | 1 |
| Pakistan | 3 |
| Papua New Guinea | 1 |
| Paraguay | 2 |
| Peru | 4 |
| Philippines | 6 |
| Romania | 9 |
| Russia | 2 |
| Rwanda | 3 |
| Samoa | 1 |
| Sao Tome and Principe | 2 |
| Senegal | 1 |
| Serbia | 9 |
| Sierra Leone | 2 |
| Somalia | 4 |
| South Africa | 7 |
| South Sudan | 1 |
| Sri Lanka | 1 |
| Sudan | 7 |
| Swaziland | 2 |
| Syria | 1 |
| Tajikistan | 2 |
| Tanzania | 3 |
| Thailand | 4 |
| Timor-Leste | 2 |
| Togo | 2 |
| Tunisia | 1 |
| Turkey | 3 |
| Uganda | 4 |
| Ukraine | 3 |
| Uzbekistan | 1 |
| Vanuatu | 2 |
| Venezuela | 1 |
| Vietnam | 2 |
| Yemen | 5 |
| Zambia | 6 |
| Zimbabwe | 1 |
| Total | 352 |

**Table 7 (ST7): Countries covered by TrACSS and GSEAR**

|  | LMICs not covered by GSEAR | Additional LMICs covered by GSEAR (Not TrACSS) | LMICs not covered by both GSEAR and TrACSS |
| --- | --- | --- | --- |
| 1 | Algeria | Angola | American Samoa |
| 2 | American Samoa | Bosnia & Herzegovina | Dominica |
| 3 | Djibouti | Comoros | El Salvador |
| 4 | Dominica | Congo | Guyana |
| 5 | El Salvador | Gambia | Nauru |
| 6 | Fiji | Grenada | Suriname |
| 7 | Guyana | Guatemala | Tonga |
| 8 | Democratic People's Republic of Korea | Guinea-Bissau | Tuvalu |
| 9 | Liberia | Haiti |  |
| 10 | Mauritania | Jamaica |  |
| 11 | Micronesia | Kiribati |  |
| 12 | Nauru | Kosovo |  |
| 13 | Niger | Marshall Islands |  |
| 14 | Solomon Islands | Samoa |  |
| 15 | St. Lucia | Sao Tome and Principe |  |
| 16 | St. Vincent and the Grenadines | Venezuela |  |
| 17 | Suriname |  |  |
| 18 | Tonga |  |  |
| 19 | Turkmenistan |  |  |
| 20 | Tuvalu |  |  |

**Supplementary Figure 1 (SF1): Comparison of TrACSS (AMR Country Self-assessment survey) and Global Expert Survey on Antimicrobial Resistance (GSEAR) (Pie Chart)**

|  |  |
| --- | --- |
|  |  |
| Fig 1: Comparison of TrACSS (AMR Country Self-assessment survey) and Global Expert Survey on Antimicrobial Resistance (GSEAR) | |

**Supplementary Figure 2 (SF2): Comparison of TrACSS (AMR Country Self-assessment survey) and Global Expert Survey on Antimicrobial Resistance (GSEAR) results (Bar chart)**

| **A. The existence of a National Action Plan on AMR** | **B. The presence of legislative policies restricting antibiotic use** |
| --- | --- |
|  |  |
|  |  |
| **C. National implementation of training programs for health professionals** | **D. National monitoring system for**  **use of antimicrobials** |
|  |  |
| Fig 2: Comparison of TrACSS (AMR Country Self-assessment survey) and Global Expert Survey on Antimicrobial Resistance (GSEAR) | |
